# IL-1β Promotes TGF-β1 and IL-2 Dependent Foxp3 Expression in Regulatory T Cells

**DOI:** 10.1371/journal.pone.0021949

**Published:** 2011-07-11

**Authors:** Balaji B. Ganesh, Palash Bhattacharya, Anupama Gopisetty, Jianrong Sheng, Chenthamarakshan Vasu, Bellur S. Prabhakar

**Affiliations:** 1 Departments of Microbiology and Immunology, College of Medicine, University of Illinois at Chicago, Chicago, Illinois, United States of America; 2 Department of Surgery, College of Medicine, University of Illinois at Chicago, Chicago, Illinois, United States of America; New York University, United States of America

## Abstract

Earlier, we have shown that GM-CSF-exposed CD8α− DCs that express low levels of pro-inflammatory cytokines IL-12 and IL-1β can induce Foxp3+ Tregs leading to suppression of autoimmunity. Here, we examined the differential effects of IL-12 and IL-1β on Foxp3 expression in T cells when activated in the presence and absence of DCs. Exogenous IL-12 abolished, but IL-1β enhanced, the ability of GM-CSF-exposed tolerogenic DCs to promote Foxp3 expression. Pre-exposure of DCs to IL-1β and IL-12 had only a modest effect on Foxp3− expressing T cells; however, T cells activated in the absence of DCs but in the presence of IL-1β or IL-12 showed highly significant increase and decrease in Foxp3+ T cell frequencies respectively suggesting direct effects of these cytokines on T cells and a role for IL-1β in promoting Foxp3 expression. Importantly, purified CD4+CD25+ cells showed a significantly higher ability to maintain Foxp3 expression when activated in the presence of IL-1β. Further analyses showed that the ability of IL-1β to maintain Foxp3 expression in CD25+ T cells was dependent on TGF-β1 and IL-2 expression in Foxp3+Tregs and CD25− effectors T cells respectively. Exposure of CD4+CD25+ T cells to IL-1β enhanced their ability to suppress effector T cell response in vitro and ongoing experimental autoimmune thyroidits in vivo. These results show that IL-1β can help enhance/maintain Tregs, which may play an important role in maintaining peripheral tolerance during inflammation to prevent and/or suppress autoimmunity.

## Introduction

Cytokines play a crucial role in regulating the immune response in that they help determine if a response to an immunogenic stimulus leads to a potent effector response or the induction of immune tolerance. While cytokines such as IL-10 and TGF-β have been implicated in the induction and maintenance of peripheral tolerance [1], [2], [3], [4], IL-1β, IL-12, IL-6, IL-17, TNF-α and IFN-γ have been generally identified with inflammatory and autoimmune responses [5], [6], [7], [8], [9], [10]. These cytokines are secreted by different types of immune cells including dendritic cells (DCs). The nature of the immune response initiated by the DCs is dependent on a number of factors including the types of cytokines they produce, their maturation status, and the nature of the antigen they present [11], [12], [13], [14], [15], [16]. Immature and semi-mature DCs and anti-inflammatory cytokines produced by them promote peripheral tolerance through regulatory T cells (Tregs) [17], [18], [19].

Tregs are subpopulations of T lymphocytes that express the transcription factor Foxp3 and they help maintain immunological self-tolerance in the periphery and prevent autoimmunity. Tregs are divided into two types: naturally occurring Tregs derived from thymus (*nTregs*) and Tregs induced in the periphery (*induced or adaptive Tregs*) [20], [21], [22]. Although the mechanism of induction of Foxp3+ T cells and maintenance of thymus derived nTreg function in the periphery have not been completely elucidated, a critical role for cytokines such as TGF-β1 has been implicated [3], [23], [24], [25]. Recent studies have confirmed a role for IL-2 in the development, homeostasis as well as effective functioning of Tregs [26], [27]. In addition, non-activated and/or partially activated DCs can help maintain effective Treg function in the periphery [18], [28], [29]. On the other hand, pro-inflammatory cytokines and highly activated DCs are known to abrogate the function of both populations of Tregs leading to breakdown of self-tolerance and induction of autoimmunity [5], [8], [9], [30], [31].

DCs that produce significantly low levels of inflammatory cytokines and/or higher amounts of anti-inflammatory cytokines are known to induce and/or expand Foxp3+ Tregs in the periphery [32], [33], [34]. In our earlier studies we showed that DCs of mice that were treated with GM-CSF were resistant to maturation even when exposed to inflammatory agents such as complete Freund's adjuvant (CFA) and maintain semi-mature tolerogenic phenotype, induce Tregs, and suppress autoimmunity upon immunization with self-antigens [19], [35], [36], [37]. Further studies have shown that this tolerogenic effect of GM-CSF exposed DCs on T cells is associated with suppressed levels of inflammatory cytokines such as IL-1β and IL-12 [19], [37], [38]. Therefore, the current study was initiated to understand the relative importance of IL-1β and IL-12 in influencing Foxp3+ Treg induction and/or expansion upon T cell activation in the presence of GM-CSF exposed tolerogenic DCs. Our results show that IL-1β, which has been hitherto considered a pro-inflammatory cytokine, could promote Foxp3+ expression in activated T cells. On the other hand, IL-12 abrogated Foxp3 induction in activated T cells. Further analyses showed that IL-1β can exert an adjuvant effect on TGF-β1 and IL-2 dependent maintenance of Foxp3+ Tregs. Collectively, our results show a unique role for IL-1β in promoting and maintaining Treg function.

## Materials and Methods

### Mice

Six- to 8-week-old female CBA/J, CD45.1 and wild type C57/B6 mice were purchased from the Jackson Laboratory (Bar Harbor, ME). Foxp3-GFP KI mice (CD45.2) were kindly provided by Dr. Vijay Kuchroo, Harvard Institutes of Medicine. Mice were housed and bred in the College of Medicine Research building at the University of Illinois (Chicago, IL) and provided food and water *ad libitum*. Animals were cared for in accordance with the University of Illinois Animal Care and Use Committee guidelines.

### GM-CSF & Abs

Recombinant mouse-GM-CSF was purchased from Invitrogen (Carlsbad, CA). The FITC or APC-conjugated anti-CD11c (N4181), CD8α (53-6.7), CD25 (3C7), CD80 (16-10A1), CD86 (GL-1) and CD40 (3/23), FITC-labeled CD4, streptavidin PE, isotype control monoclonal Abs, biotinylated anti-TGF-β Ab (A75-3), and purified anti-CD16/CD32R Ab (2.4G2), as Fc receptor block, were purchased from BD Biosciences Pharmingen (San Diego, CA). APC or EF anti-Foxp3 (FJK-16S), PE- anti IL2 (JES6-5H4), PE anti-CD4 (GK1.5), PE anti-IL-10 (JES5-16E3), APC anti-IL-17 (ebio17B7) Abs were obtained from eBioscience (San Diego, CA). CFSE was purchased from Invitrogen and neutralizing Abs to TGF-β (1D11), IL-2 (MAB702), IL1R1 (JAMA147), anti-IL-12 (polyclonal), were purchased from R&D Systems (Minneapolis, MN).

### T cells, DCs and mouse thyroglobulin

CD4+25+ and CD4+CD25− T cells were purified from spleen by positive and negative selection, respectively, using Abs conjugated to magnetic beads (Miltenyi Biotec, Auburn, CA). For isolation of Foxp3+GFP+ and Foxp3-GFP- cells, CD4+ T cells were first isolated from spleens of eGFP-Foxp3 knock in mice using negative selection kit (Miltenyi Biotec) followed by cell sorting for GFP+ and GFP- cells using the MoFlo cell sorter (DakoCytomation). For some experiments, these cells were stained with PE-labeled anti-CD25 Abs and further sorted based on their expression of GFP and CD25. CD11c+ DCs were first isolated from spleens by positive selection using anti-CD11c Ab-labeled magnetic beads, and CD8α+ and CD8α− DCs were further purified using the MoFlo cell sorter after staining with fluorochrome-labeled anti-CD11c and anti-CD8α Abs. Normal mouse thyroids were obtained from BiochemMed (Winchester, VA) or were isolated from mice during necropsy and the mTg was prepared as described earlier [39].

### GM-CSF treatment and immunization with thyroglobulin

Mice were given intra-peritoneal (i.p.) injections of GM-CSF (2 µg/mouse/day) for 5 consecutive days, from days 1–5 and 15–19. Control mice received PBS. Mice were immunized subcutaneously (s.c.) with mTg (100 µg/mouse) emulsified in complete Freund's adjuvant (CFA, Sigma) on days 6 and 20.

### Effects of GM-CSF treatment on DC maturation

GM-CSF-treated and control mice were sacrificed 48 h after mTg immunization. Purified CD8α+ and CD8α− DCs were obtained from spleens and RNA for RT-PCR was isolated from these purified DC populations using Trizol (Invitrogen). Cytokine transcript levels for IL-10, IL-6, TNF-α, IL-1β, and IL-12 were determined by RT-PCR using a commercial kit following manufacturer's guidelines (Maxim Biotech, Rockville, MD.).

### IL-12 and IL-1β treatment of DCs

Isolated splenic CD11c+CD8α− DCs were treated with IL-1β or IL-12 (e-bioscience) for 24 hours. These cells were then stained with fluorochrome-labeled anti-mouse CD11c Ab in combination with anti-mouse CD80, CD86 or CD40 Abs and analyzed using a flow cytometer (Cyan; DakoCytomation). Supernatants from these cultures were tested for cytokines by ELISA. For some experiments, these DCs were washed and cultured with T cells in antigen-presentation assays.

### In vitro cell culture assay

Purified total CD4+ T cells, or sorted CD4+25+ or CD4+25− T cells (4×10^4^ cells/well) were cultured with or without the addition of soluble anti-CD3 and anti-CD28. For some experiments, CD4+GFP+ and CD4+GFP− T cells isolated from GFP-KI mice were used. For certain co-culture experiments, CD4+GFP+ or CD4+GFP− T cells of CD45.2 mice were co-cultured with CD4+CD25− or CD4+CD25+ T cells from CD4+CD45.1 T cells in the presence of anti-CD3 and anti-CD28 Abs. In some experiments, CD4+ T cells were cultured with DCs either in the presence of IL-1β or DCs that had been previously exposed to IL-1β. In other experiments the T cells were co-cultured in the presence of GMCD8α− DCs or ConCD8α− DCs at different ratios. Twenty four hours later, medium, IL-12 or IL-1β, or both were added at concentrations specified in the legend. After a total of 5 days in culture, cells were analyzed by flow cytometry. In some experiments, these cells were stained with CFSE and assessed for their ability to proliferate. For the 3H-TdR-based proliferation assays, cells were pulsed for the last 18 h of culture with 1 µCi 3H-thymidine after which they were harvested and assessed for thymidine incorporation. In some experiments, neutralizing Abs to TGF-β, IL-12 or IL-1β were added to the cultures at specified concentrations. All results are expressed as the mean cpm ±SD of triplicate cultures. Cell-free supernatants were collected for cytokine analysis by ELISA.

### Measurement of cytokine production

Cell free supernatants were assayed for IFN-γ, IL-2, IL-10, TGF-β, IL-12 and IL-1β by sandwich ELISA using paired Abs following the manufacturer's instructions (eBioscience). The OD values were determined at 450 nm using a microplate reader (Bio-Rad). Cytokine levels were determined using corresponding cytokine standards.

### Evaluation of EAT

Thyroids were fixed in formalin, embedded in paraffin, sectioned across both lobes and stained with H&E. Thyroid pathology was evaluated and the extent of thyroid lymphocytic infiltration, as a marker of disease severity, was scored using a scale of 1+ to 5+. An infiltrate of at least 125 cells in one or several foci was scored 1+. Ten to twenty foci of cellular infiltration involving up to 25% of the gland was scored 2+. An infiltration involving up to 25–50% of the gland was scored 3+. Destruction of greater than 50% of the gland was scored 4+, and near complete destruction of the gland with very few or no remaining follicles was scored 5+. Thyroids were evaluated and scored in a blinded fashion.

### Statistical analysis

Mean, standard deviation, and statistical significance were calculated using the SPSS application software. Statistical significance was determined using the non-parametric paired t test. In most cases, values of individual treated and/or immunized groups were compared with that of untreated and/or non-immunized group unless mentioned otherwise. In studies comparing more than two groups, one-way analysis of variance was used to determine p values and assess significance. A p value of <0.05 was considered significant.

## Results

### IL-1 β and IL-12 produce contrasting effects on Foxp3 expression in activated T cells

Earlier, we have shown that mice treated with GM-CSF carry large numbers of CD8α− tolerogenic DCs with the ability to induce and/or expand antigen specific Foxp3+ Tregs and suppress autoimmunity [19]. We have also reported that CD8α− DCs from GM-CSF treated mice produce significantly lower amounts of pro-inflammatory cytokines IL-1β and IL-12 compared to their counterparts from control mice when exposed to inflammatory agents such as CFA [19], [37], [38] indicating that suppressed levels of these cytokines have a positive effect on the ability of DCs to induce Foxp3 expression in T cells upon activation. Hence, the influence of IL-1β and IL-12 during T cell activation by DCs in terms of Foxp3 expression in T cells was examined.

**Figure 1:**
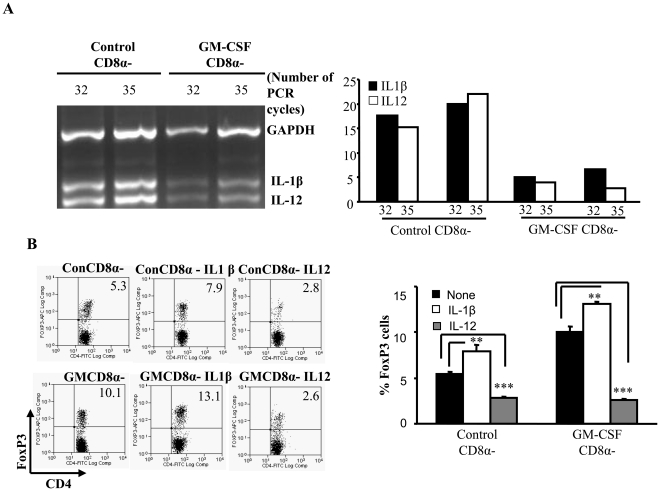
GM-CSF-treated DCs show suppressed levels of IL-12 and IL-1β; addition of IL-1β enhances Foxp3 expression. **A,** CBA/J mice were treated with either GM-CSF or PBS for 5 consecutive days starting on days 1 and 15. Mice were immunized twice with mTg (100 µg/100 µl) and CFA on days 6 and 20. Two days after the 2^nd^ immunization, mice were sacrificed and CD11c+ DCs were isolated from spleens by positive selection using CD11c-labeled magnetic beads. CD11c+CD8α+ and CD11c+CD8α− DCs were further purified using the MoFlo cell sorter (Beckman Coulter). RNA was isolated from GMCD8α− DCs and ConCD8α− DCs and used in a RT-PCR assay to detect cytokine transcripts with a multiplex PCR kit (Maxim Biotech). PCR products were collected at 32 and 35 cycles and were resolved on a 2% agarose gel. Densitometry analysis was performed and band densities represented as ratios of densities relative to the GAPDH bands from the corresponding cells are represented in the bar graph. The numbers on the X axis denotes the number of PCR cycles. ***B*** **,** CD4+ T cells were isolated from spleens of mTg-immunized CBA/J mice and cultured in the presence of DCs from GM-CSF treated mice (GMCD8α− DCs) or PBS treated control (ConCD8α−) mice (4×10^4^ cells/well) at a 1∶1 ratio in 96-well round bottom plates in the presence of mTg (100 µg/ml). Some wells received 5 ng/ml of IL-1β or IL-12, or both. Controls received medium alone. After 5 days in culture, the cells were collected and stained for Foxp3 expression along with CD4 and analyzed by flow cytometry. Representative scatter-plots of CD4+Foxp3+ cells from three independent experiments carried in triplicates, and bar graphs depicting the mean and SD values of the percentage of Foxp3+ T cells are shown. [GMCD8α− or ConCD8α− = Cultures of GM-CSF treated CD8α− DCs or Control CD8α− DCs respectively with CD4+ T cells from mTg-primed mice that received medium alone, IL-1β or IL-12.]. One-way analysis of variance was used to determine statistical significance. * p<0.05, ** p<0.01, *** p<0.001.

Consistent with our earlier reports, DCs from GM-CSF treated mice expressed considerably lower levels of IL-1β and IL-12 compared to DCs from untreated mice (Fig. 1A). Mouse thyroglobulin (mTg) pulsed DCs from GM-CSF treated and control mice were cultured with purified T cells from mTg primed-mice with or without IL-12 and/or IL-1β. As seen in Figure 1*B*, IL-12 supplemented culture had significantly lower numbers of Foxp3+ T cells compared to control wells irrespective of the type of DCs used (Fig. 1*B*). This suggested that suppressed levels of IL-12 might be contributing to the ability of GM-CSF exposed DCs to induce/expand Foxp3+ T cells. However, addition of IL-1β to these antigen presentation assay wells resulted in a significant increase in frequencies of Foxp3+ T cells over controls that did not receive any exogenous cytokine (Fig. 1*B*, P<0.01). These results suggested that suppressed IL-12, but not IL-1β, production by GM-CSF-exposed DCs conferred tolerogenic properties to DCs, which can be further enhanced by IL-1β. In addition, these results showed that the effects of exogenous IL-12 and IL-1β are similar on both the control and the GM-CSF exposed DCs in terms of their ability to influence Foxp3 expression in T cells. Hence the rest of the study was carried using control DCs to understand the differential effects of these cytokines.

### Neither IL-12 nor IL-1β treatment significantly alters the phenotype and antigen presenting properties of DCs

**Figure 2:**
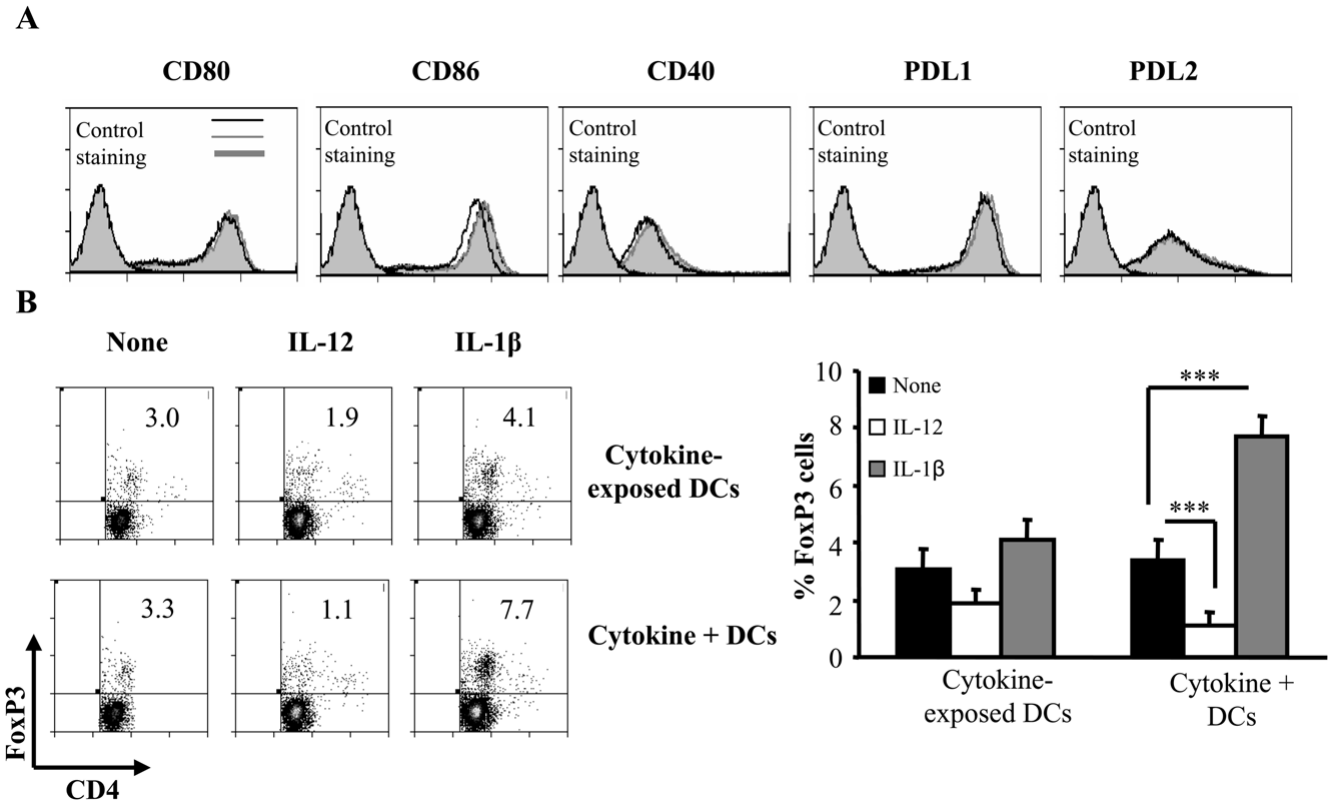
IL-12 and IL-1β act primarily on CD4+T cells to induce Foxp3. **A,** Purified CD11c+ DCs were stained with fluorochrome conjugated anti-CD11c and anti-CD8α antibodies and sorted into CD8α+ and CD8α− sub-populations. The sorted CD8α− population was cultured in the presence of 5 ng of IL-1β, IL-12 or medium alone for 24 hours and subsequently stained with anti-CD80, anti-CD86, anti-CD40, anti PDL-1 or anti-PDL2 antibodies and analyzed by flow cytometry. The percentage of cells positive for the expression of the respective co-stimulatory molecule is represented as histograms. [Light grey filled peaks towards the left represent the isotype controls and are marked. Black lines represent controls that received medium alone, Dark grey lines represent cultures that received IL-1β and grey-filled peaks indicate cultures that received IL-12]. ***B*** **,** For co-culture experiments, sorted CD8α− DCs were cultured in the presence of IL-1β or IL-12 for 24 hours, DCs were washed and then cultured with CD4+ T cells and stimulated in the presence of anti-CD3 antibody (Top row of panels). After 4 days, cells were assayed for Foxp3 expression along with CD4 using corresponding fluorochrome-labeled antibodies by flow cytometry. In parallel cultures, the CD8α− DCs were cultured in the presence of CD4+ T cells in the presence of anti-CD3 (2 µg/ml) and IL-1β was added to the DC-T cell co-cultures (Lower row of panels). Foxp3 expression was assayed as described above. Representative scatter-plots of CD4+Foxp3+ cells from two independent experiments carried out in triplicates and mean and SD of the percentages of Foxp3 expression are shown. One-way analysis of variance was used to determine statistical significance. * p<0.05, ** p<0.01, *** p<0.001.

From the above described experiment it was apparent that exogenous IL-12 and IL-1β could influence the ability of both control and GM-CSF-exposed DC's to affect Foxp3 expression in T cells suggesting that the effects of these cytokines may not be on the DCs themselves but primarily on T cells. In order to confirm this and to examine if the cytokines secreted by DCs have an autocrine/paracrine effect on the activation state of DCs that can in turn influence the T cell function, we examined CD8α− DCs that were cultured with and without IL-12 and IL-1β for expression levels of co-stimulatory molecules. Fig. 2*A* shows that surface expression levels of co-stimulatory molecules CD80, CD86, CD40, PDL-1 and PDL-2 remained largely unaltered after treatment with either cytokine. In addition, IL-12 and IL-1β- treated DCs showed similar cytokine profiles to that of untreated DCs (not shown). To test the ability of cytokine-exposed DCs to influence Foxp3 expression in T cells, CD4+ T cells were activated using anti-CD3 Ab in the presence of these DCs and examined for Foxp3 expression. As observed in Fig. 2*B*, continuous presence of these cytokines in DC:T cell cultures, but not exposure of DCs to cytokines prior to their addition to the culture, produced significant effect on the ability of T cells to express Foxp3. T cell:DC cultures, in which IL-1β was continuously present, showed a significantly higher number of Foxp3+ T cells compared to cultures where cytokine pre-exposed DCs were used. On the other hand, significantly fewer numbers of T cells activated in the presence of DCs and IL-12 expressed Foxp3 compared to T cells activated in the presence of IL-12 pre-exposed DCs. The expression of the IL-1β receptor (IL-1R2) was however detected on both DCs as well as T cells with and without treatment with IL-1β (Supporting figure 1). These observations strongly indicated that the primary and direct effects of IL-1β and IL-12 are on T cells.

### IL-1β acts directly on T cells and affects Foxp3 and TGF-β1 expression in them in a concentration dependent manner

**Figure 3:**
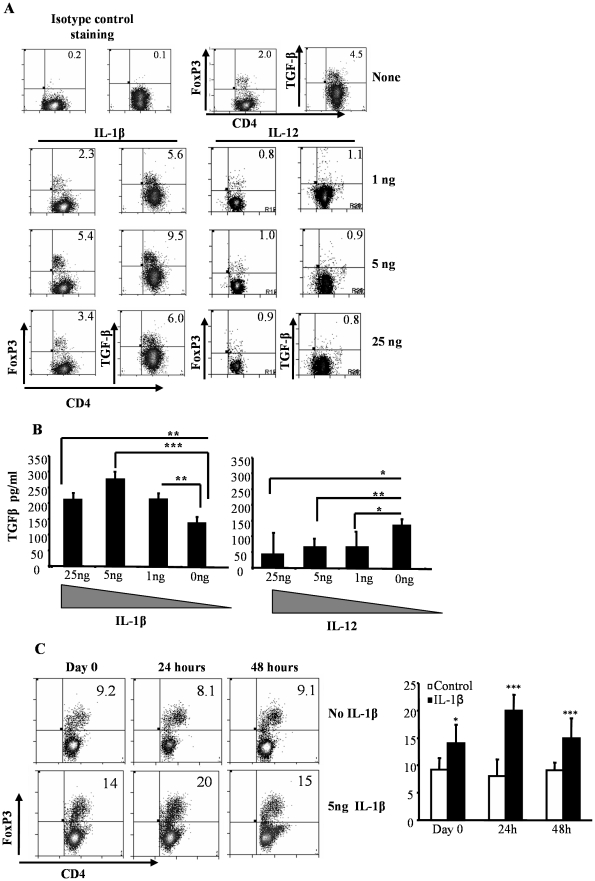
The effects of IL-1β on CD4+ T cells is dose-dependent and results in increased TGF-β1 production. **A,** CD4+ T cells were isolated from the splenocytes of wild type C57Bl/6. 4×10^4^ CD4+ T cells/well cultured in 96-well round bottom plates in the presence of 2 µg/ml of anti-CD3 and 0.8 µg/ml of anti-CD28. Twenty four hours later, cell cultures received different concentrations of IL-1β, IL-12, or medium as mentioned. Four days later, CD4+T cells were re-stimulated with anti-CD3 and anti-CD28 in the presence of monensin for 6 hours to prevent cytokine secretion. Cells were subsequently analyzed for Foxp3 and TGF-β1 expression along with CD4 by flow cytometry. Representative scatter-plots of similar results from 3 independent experiments are shown. These values were compared with cultures that received medium, and t-test was used to determine statistical differences. p<0.05 was considered significant. ***B*** TGF-β1 levels in supernatants from cultures shown in Fig-3*A* were quantified by ELISA. ***C*** **,** 4×10^4^ CD4+ T cells/well cultured in 96-well round bottom plates in the presence of 2 µg/ml of anti-CD3 and 0.8 µg/ml of anti-CD28. IL-1β was added to these cultures either at the time of culture or twenty four hours later, cell cultures received 5 ng of IL-1β or medium as mentioned. Four days later, CD4+T cells were subsequently analyzed for Foxp3 expression along with CD4 by flow cytometry. Results shown as a bar graph are mean+SD values and representative one of three independent experiments carried out in triplicates. One-way analysis of variance was used to determine statistical significance. * p<0.05, ** p<0.01, *** p<0.001.

To further confirm the direct effect of IL-1β and IL-12 on T cells in terms of Foxp3 expression, purified CD4+ T cells were activated using anti-CD3 and CD28 Abs in the absence of DCs, but in the presence of varying amounts of these cytokines. As observed in Fig. 3*A*, the frequency of T cells that expressed Foxp3 was highest when they were activated in the presence of an optimum amount of (5 ng/ml) IL-1β. In contrast, a majority of T cells lost Foxp3 expression when they were activated in the presence of IL-12 irrespective of the concentration of this cytokine in the culture (lower right panel). Of note, this effect of IL-12 on Foxp3 expression in T cells was persistent even at picogram concentrations (data not shown). These observations further demonstrate that IL-1β and IL-12 act directly on T cells and influence Foxp3 expression in them.

Addition of optimum amount of IL-1β to T cell activation cultures also resulted in a significant increase in the frequency of TGF-β expressing T cells (Fig. 3*A*, lower left panel) compared to T cells activated in the absence of exogenous IL-1β (Fig. 3*A*, upper panel). In contrast, when IL-12 was added to the culture, there was a significant decrease in the TGF-β1+ T cell frequency in the culture as compared to controls. Furthermore, the amounts of TGF-β1 secreted in the culture supernatants were significantly higher in IL-1β, but lower in IL-12 supplemented T cell activation cultures (Fig. 3*B*). Addition of IL-1β 24 hours after activation yielded the highest percentage of Foxp3+ T cells when compared to cultures that received no IL-1β or received it on day 0 or 48 hours after activation (Fig. 3*C*). Although, the exact mechanisms involved in the increase in Foxp3+ T cells that received IL-1β 24 h after activation is not clearly known, we speculate that this may be related to the Foxp3 promoter occupancy and therefore unavailable for gene transcription in resting T cells. Activation of T cells may cause the promoter to be ‘unblocked’ causing the Foxp3 gene to become available for modulation by IL-1β. These observations indicated that IL-1β and IL-12 can directly act on T cells upon activation and modulate Foxp3+ and TGF-β1 expression. Since earlier studies have implicated TGF-β in the induction of Tregs [3], [4], the above data suggested that the changes in the Foxp3+ T cell frequencies when the T cell cultures were supplemented with IL-1β and IL-12 could be a consequence of altered levels of secreted TGF-β1. These observations also suggested that optimal concentrations of IL-1β can promote Treg function. Hence the effects of IL-1β on Tregs in terms of Foxp3 expression and suppressor function were examined as described below.

### IL-1β helps maintenance of Foxp3 expression in CD4+CD25+ T cells upon activation

**Figure 4:**
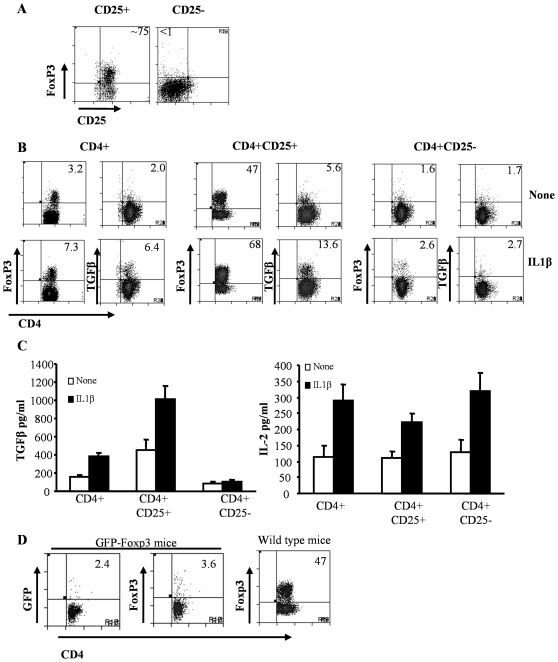
The primary effect of IL-1β is on CD4+CD25+ T cells. CD4+25+ and CD4+CD25− T cells were purified from spleens of naïve C57Bl/6 mice by positive and negative selection respectively, using antibodies bound to magnetic beads. Briefly, CD4+ T cells were isolated by negative selection using magnetic beads. CD25+ and CD25− T cells were isolated from the CD4+ populations by positive and negative selection respectively. Purified CD4+, CD25+ and CD25− T cells were stimulated with 2 µg/ml of anti-CD3 and 0.8 µg/ml of anti-CD28. Twenty four hours later, cell cultures received 5 ng/ml of IL-1β, or medium as control. ***A,*** Purified CD4+CD25− T cells and CD4+CD25+ T cells were stained for Foxp3 ***B,*** Four days after the addition of cytokines, cells were analyzed for Foxp3 and TGF-β1 expression along with CD4 by flow cytometry. ***C,*** For cytokine analysis, supernatants from the co-cultures were collected and assayed for TGF-β1 and IL-2. ***D,*** CD25+ T cells and GFP+ (Foxp3+) T cells were isolated from wild type C57Bl/6 and Foxp3-GFP KI mice respectively and were stimulated with 2 µg/ml of anti-CD3 and 0.8 µg/ml of anti-CD28. Four days later, cells were analyzed for Foxp3 or GFP expression along with CD4 by flow cytometry T-test was used to determine statistical differences [None = cultures that received medium alone and IL-1β = cultures that received IL-1β].

To examine whether IL-1β acts on natural Foxp3+ Tregs and causes their expansion or induces Foxp3 expression in effector T cells upon activation, CD4+CD25+ and CD4+CD25− T cells were enriched from naive mice (Fig. 4*A*) and activated using anti-CD3 and anti-CD28 Abs in the presence of optimum concentration of IL-1β . Consistent with the observations shown in Fig. 3, significantly higher number of total CD4+ T cells activated in the presence IL-1β expressed Foxp3 and TGF-β1 compared to those cells activated without IL-1β (Fig. 4*B*; left panels). Notably, only about 50% of enriched CD25+ T cells expressed Foxp3 after 4 days of activation *in vitro* (Fig. 4*B*; middle panels). The reduction seen in Foxp3 levels in CD25+ (i.e. from 75% to 47% without IL-1β and 68% with IL-1β; see panel 4A and 4B;) as well as in CD4+ cultures (Figs. 3 and 4) suggested that a significant number of CD4+CD25+ nTregs lost their Foxp3 expression and/or Foxp3− cells in enriched CD4+CD25+ population expanded selectively during activation. Nevertheless, a significantly higher proportion of CD4+CD25+ T cells activated in the presence of IL-1β expressed both Foxp3 and TGF-β1 compared to their respective controls. This observation suggested that IL-1β signaling may be contributing to the maintenance of Foxp3 expression in nTregs and/or promoting Foxp3 expression in activated effector T cells with the help of TGF-β1. Importantly, CD4+CD25− T cells activated in the presence of IL-1β failed to show a significant increase in Foxp3+ and TGF-β1+ T cell frequencies compared to their control counterparts (Fig. 4*B*; right panels). This suggested that the effect of IL-1β, in terms of Foxp3 and TGF-β1 expression, is primarily on nTregs. Examination of supernatants from the above cultures revealed that T cells activated in the presence of IL-1β produced significantly higher amounts of not only TGF-β1, but also IL-2, compared to cells activated without IL-1β (Fig. 4*C*). Interestingly, while CD4+CD25+ population, but not CD4+CD25− population produced large amounts of TGF-β1 when activated in the presence of IL-1β, both populations produced significantly higher amounts of IL-2 in the presence of IL-1β. CD4+CD25+ T cells had higher basal levels of IL-2 than expected. This is probably due to either the presence of CD25+Foxp3− activated T cells in the culture or due to IL-2 secretion by potentially contaminating CD25− T cells as CD25+ purifications using magnetic beads yielded approximately 75% CD25+ T cells. This suggested a potential role for IL-2 in IL-1β-mediated TGF-β1-dependent Foxp3 expression in T cells. Foxp3+ Tregs are known to be refractive to expansion even upon activation by anti-CD3 and anti-CD28. Therefore, to understand as to why IL-1β-treated cultures had increased Foxp3-expressing cells, we used purified Foxp3+ Tregs from the Foxp3-GFP knock-in mice by cell sorting and activated them with anti-CD3 and anti-CD28. After 4 days of culture, the cells were analyzed for both GFP as well as Foxp3 expression. The number of GFP+ T cells or Foxp3-expressing cells recovered at the end of 4 days were very few (<5% Foxp3+). In contrast, CD25+ T cells expressed much higher levels of Foxp3 (47%) (Fig. 4*D*, right panel). This apparent difference in the frequency of Foxp3+ cells was most likely due to the inability of Foxp3+ cells to survive/proliferate in the absence of IL-2, which in CD25+ cultures was likely produced by activated non-Foxp3+ T cells. Therefore, we used CD25+ T cells in our subsequent experiments.

### IL-1β mediated increase in Foxp3+ T cells is TGF-β1 and IL-2 dependent

**Figure 5:**
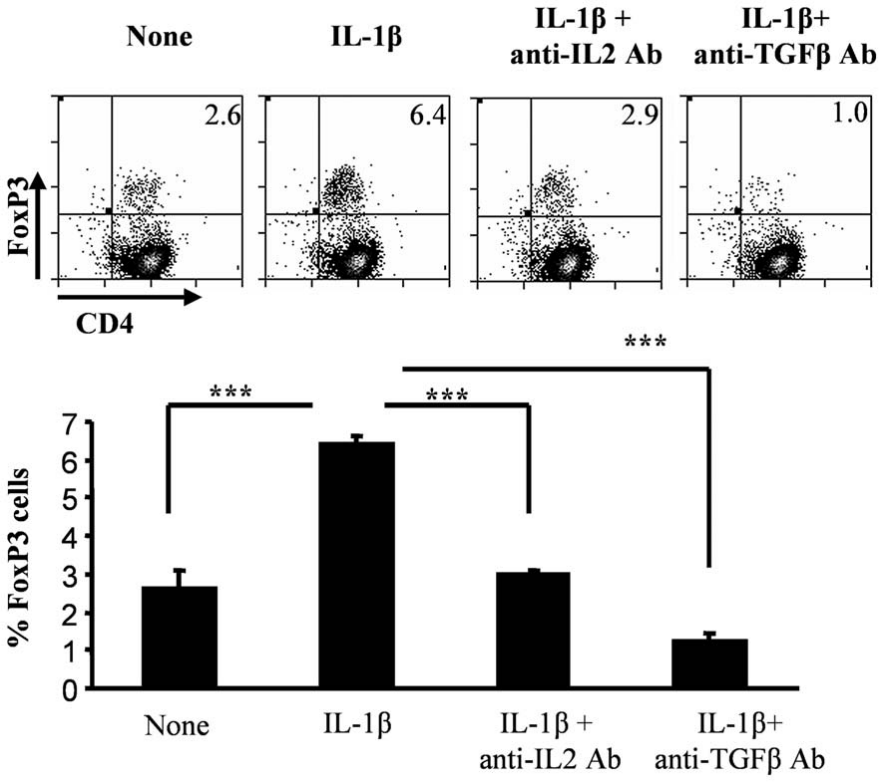
The induction of Foxp3 by IL-1β is dependent on both IL-2 and TGF-β1. CD4+ T cells that were purified from spleens of naïve C57Bl/6 mice were stained with CFSE and cultured in 96-well round bottom plates in the presence of 2 µg/ml of anti-CD3 and 0.8 µg/ml of anti-CD28. Twenty four hours later these cell cultures received isotype control antibodies (None), or 5 ng/ml of IL-1β along with isotype control antibodies (IL-1β) and served as controls. Saturating concentrations of neutralizing anti-TGF-β1 (30 µg/ml) or anti-IL-2 (20 µg/ml) were added along with IL-1β. Four days later, CD4+T cells were analyzed for Foxp3 expression along with CD4 by flow cytometry. These values were compared with values from cultures that received medium, and t-test was used to determine statistical differences. Results shown are representative of two independent experiments carried out in triplicates.

In order to determine the roles of IL-1β-induced-TGF-β and IL-2 in promoting Foxp3+ expression in T cells, purified total CD4+ T cells were activated using anti-CD3 and anti-CD28 Abs in the presence of IL-1β, and with and without neutralizing Abs to TGF-β and IL-2. Consistent with the observations from above experiments (Figs. 3 and 4), a significantly higher proportion of T cells activated in the presence of IL-1β expressed Foxp3. However, neutralization of either IL-2 or TGF-β1 using respective Abs in these cultures resulted in complete reversal of the Foxp3-inducing effect of IL-1β in CD4+ T cells (Fig. 5). These observations clearly indicated that IL-1β-mediated expression of Foxp3 in T cells requires both TGF-β and IL-2 induced signaling.

### IL-1β enhances proliferative response of Foxp3− cells, but not Foxp3+ Tregs upon activation

**Figure 6:**
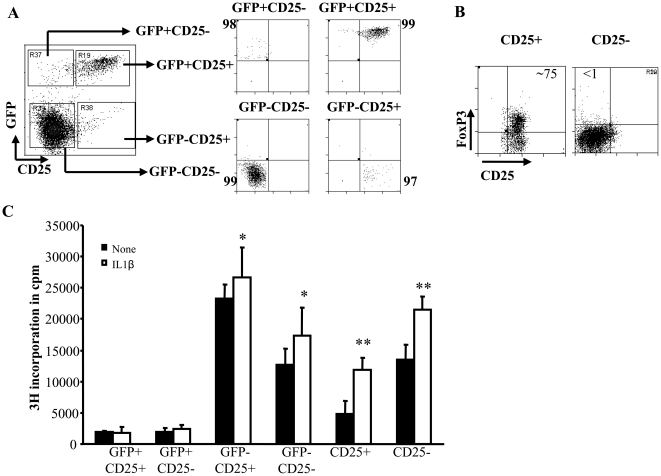
Highly purified Foxp3GFP+ Tregs do not proliferate upon treatment with IL-1β. **A,** CD4+ T cells from GFP-Foxp3 KI mice were purified from spleens by negative selection using antibodies bound to magnetic beads. These CD4+ T cells were stained with PE labeled anti-CD25 to isolate highly purified GFP+CD25+, GFP+CD25−, GFP−CD25+ and GFP−CD25− T cells. Percentage purity of sorted cells are shown. These cells (4×10^4^ cells/well) were cultured in 96-well round bottom plates in the presence of 2 µg/ml of anti-CD3 and 0.8 µg/ml of anti-CD28. CD4+CD25+ and CD4+CD25− T cells were isolated from wild type C57Bl/6 mice and were activated as described above. Twenty four hours later, cells were treated with 5 µg/ml of IL-1β, or medium for 4 days. ***B,*** Around 75% of the purified CD25+ T cells were positive for Foxp3. ***C,*** Cells were cultured for 48 h and pulsed for the last 18 h of culture with 1 µCi 3H-thymidine after which they were harvested and assessed for radioactivity. Bar graphs show the mean cpm + SD of thymidine incorporation in triplicate cultures. These values were compared with those from cultures that received medium, and t-test was used to determine statistical differences. * p<0.05, ** p<0.01, *** p<0.001.

Foxp3+ Tregs are considered anergic and are not known to proliferate upon stimulation with anti-CD3 and anti-CD28 Abs, and yet we observed significantly higher Foxp3+ T cell frequencies in IL-1β supplemented CD4+CD25+ T cell activation cultures. To determine the effect of IL-1β on sub-populations of T cells during activation, enriched Foxp3+CD25+, Foxp3−CD25+, Foxp3+CD25−, Foxp3−CD25− T cell subpopulations from Foxp3-GFP knock-in mice (Fig. 6*A*; left panel) were examined for their ability to proliferate upon activation in the presence or absence of IL-1β . CD4+CD25+ T cells and CD4+CD25− T cells from wild type mice (Fig. 6B) were also examined for their proliferative response upon activation in the presence of IL-1β. As observed in Fig. 6*C*, Foxp3+ (GFP+) T cells activated with or without IL-1β showed little or no proliferation irrespective of surface CD25 expression. In contrast, IL-1β induced a significant proliferation of Foxp3− (GFP−) T cell populations. Importantly, both CD4+CD25+GFP− and CD4+CD25−GFP− populations showed significantly higher levels of proliferation compared to Foxp3+ (i.e. GFP+) cells. In addition, both CD25+ and CD25− populations showed enhanced proliferative response when the cultures were supplemented with IL-1β. These results indicated that IL-1β promotes proliferation of CD25+ T cells, which contains both Foxp3+ and Foxp3− cells, but cannot promote proliferation of purified Foxp3+ cells. These results suggested that CD4+Foxp3− T cells may have a role in maintaining Foxp3 expression in Foxp3+Tregs during activation and expansion. These results also indicated that nTreg expansion requires additional cytokines such as IL-2 that probably comes from non-Foxp3+ cells.

### IL-1β-mediated maintenance of Foxp3 expression in Tregs is dependent on Treg derived TGF-β1 and effector T cell derived IL-2

**Figure 7:**
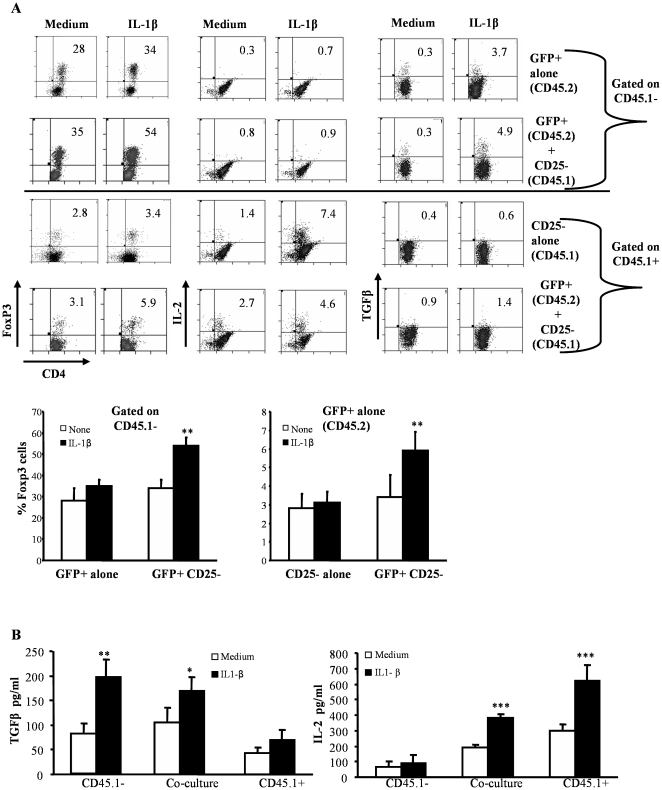
Production of IL-2 by CD25− T cells and TGF-β1 by CD25+ Tregs is essential for increased in Foxp3+ Tregs upon IL-1β treatment. **A,** CD4+ T cells were purified from spleens of naïve CD45.1+C57Bl/6 and Foxp3.GFP+CD45.2+ mice by negative selection using antibodies bound to magnetic beads. CD4+CD45.1+ T cells were further sorted into CD25+ and CD25− T cells. Similarly CD4+ T cells from GFP-KI mice were further sorted into GFP+ and GFP− T cells. CD25–CD45.1+ and Foxp3.GFP+CD45.− T cells were cultured alone or together in 96-well round bottom plates in the presence of 2 µg/ml of anti-CD3 and 0.8 µg/ml of anti-CD28. Twenty four hours later, cell cultures received 5 ng/ml of IL-1β, or medium as control. Four days later, CD4+T cells were analyzed for Foxp3, IL-2 and TGF-β+ expression after appropriately staining for those markers. The top two rows indicate the Foxp3, IL-2 and TGF-β1 expression in cells that were gated on CD4+CD45.1− populations while the bottom two rows show cells that were gated on CD4+CD45.1+ populations. Representative bar graphs show the mean + SD of of results from one of two independent experiments carried out in triplicate. ***B,*** The supernatants from the above cultures were collected and assayed for cytokines TGF-β1 and IL-2 by ELISA as described for the earlier. Results shown are mean + SD of results from one of two independent experiments carried out in triplicate.

Fig. 5 showed that IL-1β-mediated Foxp3 expression is TGF-β1 and IL-2 dependent. Consistent with earlier reports [34], [40] our above observations (Fig. 6) indicated that nTregs do not proliferate upon activation using anti-CD3 Ab likely due to lack of IL-2 production. Therefore, we examined the source of cytokines (i.e.TGF-β1 and IL-2) that contribute to Foxp3 expression in Tregs when activated in the presence of IL-1β using nTregs isolated from Foxp3−GFP-knock-in mice (CD45.2 mice) and CD4+CD25− effector T cells from wild-type mice (CD45.1 mice) (Fig. 7). When GFP+(Foxp3+) CD45.2 T cells were activated using anti-CD3 and anti-CD28 Abs, Foxp3 expression was lost in a significant proportion of cells and IL-1β failed to influence Foxp3 expression in them significantly (Fig. 7; uppermost row). Considerable number of these activated GFP+ (Foxp3+) cells expressed TGF-β1, but not IL-2. On the other hand, when CD4+CD25− CD45.1 T cells were activated, IL-1β induced the production of IL-2 by a large number of cells, but did not influence Foxp3 or TGF-β1 expression in them (Fig. 7*A*; third row). Interestingly, co-activation of GFP+(Foxp3+) CD45.2 and CD4+CD25− CD45.1 T cells in the presence of IL-1β led to Foxp3 and TGF-β1 expression in a significantly higher number of CD45.2 T cells compared to co-culture of those cells in the absence of exogenous IL-1β (Fig. 7*A*; second row). These observations suggested that Foxp3 expression in Tregs upon activation is co-dependent upon the autocrine effects of TGF-β1 and IL-2 secreted by effector T cells, while IL-1β delivers an adjuvant effect rather than directly inducing Foxp3 expression. Of note, a higher number of CD4+CD25− CD45.1 T cells expressed Foxp3 in the presence of IL-1β and GFP+(Foxp3+) CD45.2 T cells (Fig. 7*A*; lowermost row). Correspondingly, increased levels of secreted TGF-β1 and IL-2 were observed in cultures of CD45.2+GFP+ T cells and CD45.1+CD25− CD4+T cells respectively that received IL-1β as compared to untreated controls (Fig. 7*B*).

### IL-1β-exposed CD25+ T cells show superior ability to suppress effector T cell response

**Figure 8:**
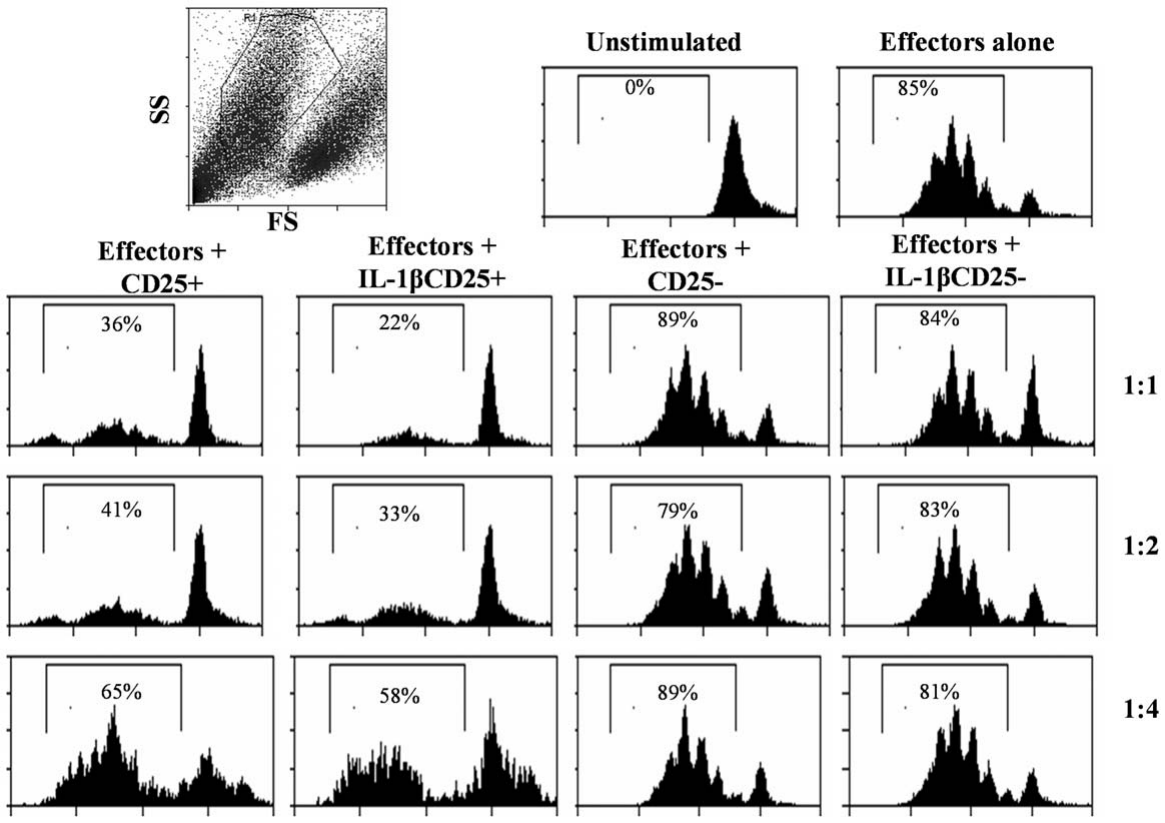
CD4+CD25+ Tregs show increased suppression of effectors upon IL-1β treatment. CD4+ T cells were purified from spleens of naïve C57Bl/6 mice using antibodies bound to magnetic beads. CD25+ T cells were further purified using magnetic beads and 2×10^5^ cells were cultured in 96-well round bottom plates in the presence of 2 µg/ml of anti-CD3 and 0.8 µg/ml of anti-CD28. Twenty four hours later, cell cultures received 5 ng/ml of IL-1β or medium (controls). Four days later, 2×10^5^ CD4+ T cells were isolated and added to CFSE-labeled CD4+CD25− effector T cells obtained from spleens of naïve mice at different effector T cell∶Treg ratio. Cultures were further stimulated with anti-CD3/anti-CD28. Irradiated splenocytes from naïve mice were used as APCs. After 4 days of culture, cell proliferation was assessed by CFSE dilution using flow cytometry. The CFSE+ population was considered 100%. Cells were gated upon the CFSE-labeled population and the percentage of CFSE^low^ cells are shown in each histogram. Results shown are the mean proliferation of CD25− effectors in triplicate cultures and are representative of two independent experiments. These values were compared with values from cultures that received medium. One way ANOVA was used to determine statistical significance. * p<0.05, ** p<0.01, *** p<0.001.

To assess the functional significance of IL-1β-mediated Foxp3 expression in T cells, CD4+CD25+ T cells that were activated using anti-CD3 and anti-CD28 Abs in the presence and absence of IL-1β were examined for their ability to suppress effector T cell proliferation in a co-culture assay. As observed in Fig. 8, both IL-1β-exposed as well as control CD25+ T cells were able to suppress the CD25− effector T cell proliferation as indicated by the CFSE dilution profile. However, significantly fewer effector T cells in the presence of IL-1β exposed CD4+CD25+ T cells showed CFSE dilution compared to effector cells activated in the presence of control CD4+CD25+ T cells at a 1∶1 ratio of Tregs and effector T cells. The differences however were no longer significant at ratios greater than 1∶2. CD25− T cells were similarly treated with or without IL-1β and cultured with effector T cells. IL-1β had no effect on the ‘suppressive’ ability of these cells. These results showed that IL-1β-exposed CD4+CD25+ T cells have a superior ability to suppress effector T cell response, perhaps due to the presence of a higher proportion of Foxp3+ and TGF-β1+ cells.

### IL-1β-exposed CD4+CD25+ T cells from mTg-primed mice suppressed mTg-specific effector T cell response

**Figure 9:**
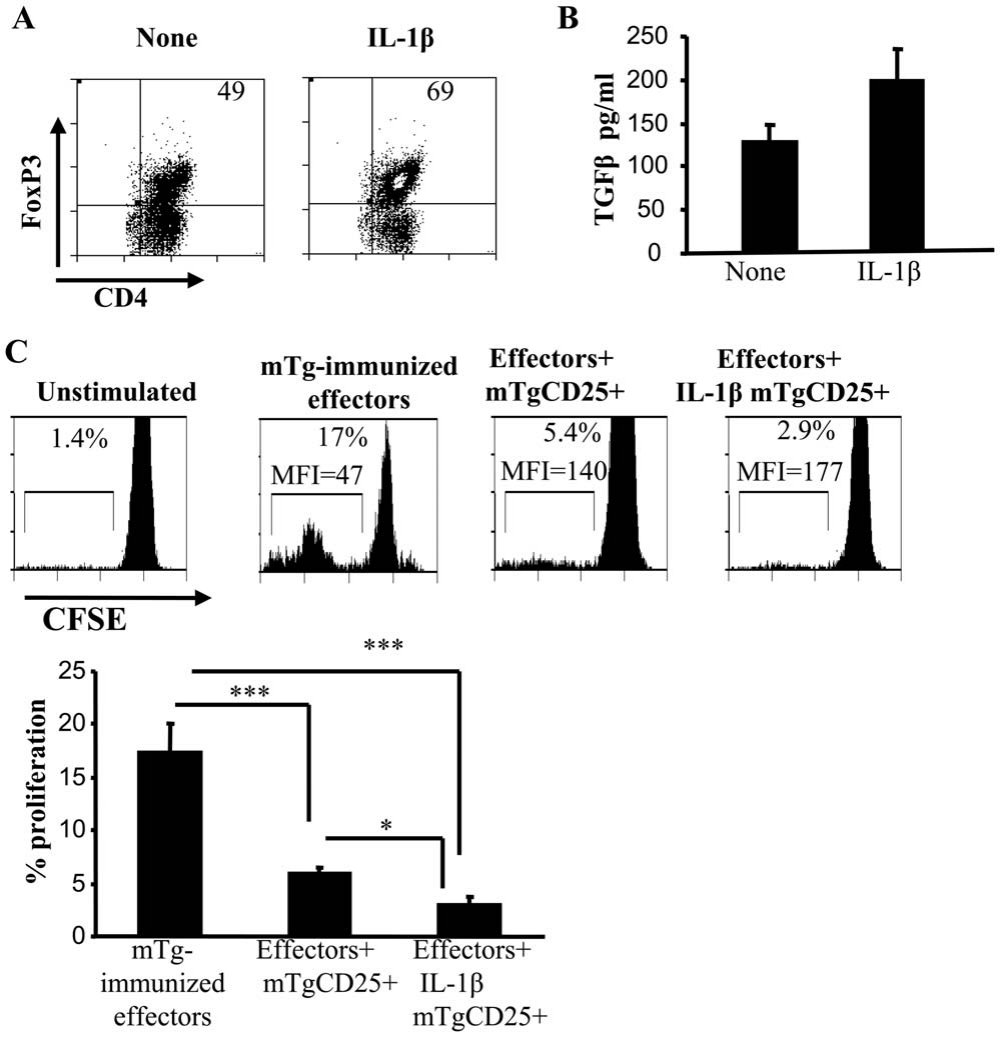
IL-1β treated Tregs show increased antigen specific suppression in vitro. CD4+CD25+ T cells were isolated from mTg-immunized mice and cultured in the presence of splenic DCs as antigen-presenting cells, IL-1β or medium and mTg (100 µg/ml). ***A,*** After 4 days, CD4+ T cells were purified and evaluated for Foxp3 expression and ***B,*** secreted TGF-β1 in supernatants by ELISA. ***C,*** These cells were added to CFSE-stained CD4+CD25− T effector cells isolated form mTg purified mice in the presence of mTg (100 µg/ml). The percentage of CFSE^low^ cells are shown in each histogram (lower panel). (mTg-immunized Teff = CD4+CD25− T cell cultures that received mTg). Bar graphs show the mean proliferation + SD of CD25− effectors in triplicate cultures. One way ANOVA was used to determine statistical significance.

In order to examine whether self-antigen specific Treg function can be enhanced with the help of IL-1β, CD4+CD25+ T cells enriched from mTg-primed CBA/J mice were activated using mTg-pulsed splenic DCs with and without IL-1β. After 4 days in culture, a significantly higher proportion of CD4+CD25+ T cells activated in the presence of IL-1β maintained Foxp3 expression (Fig. 9*A*) and produced TGF-β compared to cells that were activated without IL-1β (Fig. 9*C*) . To examine their suppressor properties, T cells from these cultures were isolated and co-cultured along with CD4+CD25− cells from mTg-primed mice and mTg-pulsed DCs. As observed in Fig. 9*C*, T cells from both IL-1β supplemented and control cultures were able to suppress mTg specific effector T cell proliferation. However, T cells from IL-1β supplemented cultures exerted superior suppressor ability on mTg-specific effector T cells compared to control T cells. These results in conjunction with that shown in Fig. 8 indicated that IL-1β helps maintain and/or enhance suppressor function of Tregs irrespective of the activation signal they receive.

### Adoptive transfer of IL-1β-induced Tregs suppress EAT

**Figure 10:**
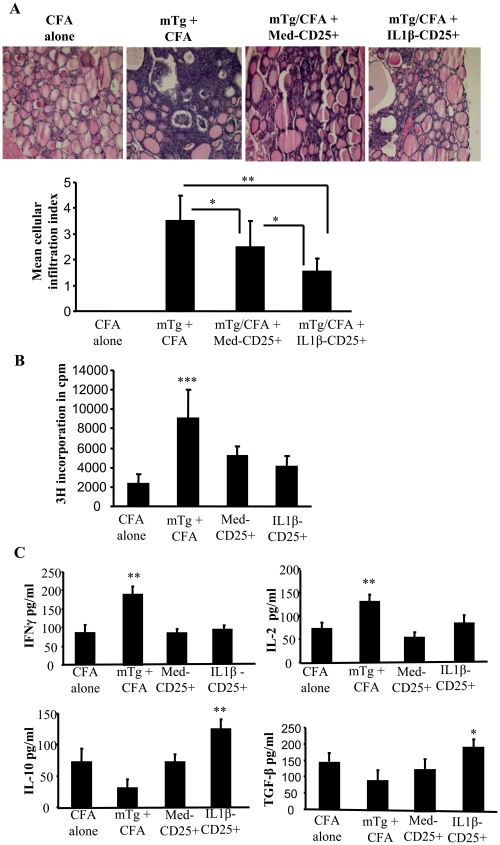
IL-1β treated Tregs from mTg-primed mice more efficiently suppress autoimmune thyroiditis. CD4+ T cells were purified from spleens of naïve CBA/J mice using antibodies bound to magnetic beads. CD25+ T cells were further purified using magnetic beads and cultured in 96-well round bottom plates in the presence of 2 µg/ml of anti-CD3 and 0.8 µg/ml of anti-CD28. Twenty four hours later, cell cultures received 5 ng/ml of IL-1β, or medium as control. After 4 days, purified CD4+CD25+ T cells (2×10^6^) from IL-1β and control group were transferred via tail vein into 7-week old naïve wild type CBA/J mice (5 mice/group). These mice were immunized with mTg emulsified in CFA, 3 and 17 days after the adoptive transfer. ***A,*** Forty days post transfer, mice were sacrificed, thyroids removed and fixed in formalin. Paraffin embedded sections were prepared and stained with H&E. Representative photomicrographs of H&E stained thyroid sections from different treatment groups are shown. Severity of thyroid lymphocytic infiltration was used as a marker of disease severity. Mean cellular infiltration index for different groups is shown in the bar graph. ***B,*** In parallel cultures, cells were pulsed for the last 18 h of culture with 1 µCi 3H-thymidine after which they were harvested and assessed for radioactivity. Bar graphs show the mean cpm + SD of thymidine incorporation in triplicate cultures. ***C*** *Ex vivo* cytokine response was estimated in splenocytes isolated from mice. Spleen cells were treated with mTg (100 µg/ml) and 3 days later supernatants were collected and estimate for cytokines IFNγ, IL-2, TGF-β1 and IL-10 by ELISA., Results shown are representative of 2 independent experiments. One way ANOVA was used to determine statistical significance. * p<0.05, ** p<0.01, *** p<0.001.

To examine the therapeutic significance of IL-1β-mediated maintenance of Foxp3 expression in Tregs and the enhancement of their suppressor function, CD4+CD25+ T cells from mTg-primed mice that were cultured with and without IL-1β as described for Fig. 9 were adoptively transferred into CBA/J mice prior to inducing experimental autoimmune thyroiditis. As anticipated, control mice that did not receive T cells developed severe thyroiditis as indicated by massive infiltration of immune cells into thyroids and follicular destruction (Fig. 10*A*). However, mice that received T cells showed less severe thyroiditis compared to non-recipient controls. Importantly, mice that received IL-1β-exposed CD4+CD25+ T cells developed less severe thyroiditis as compared to control CD4+CD25+ T cell recipients. In addition, T cells from mice that received both control and IL-1β exposed CD4+CD25+ T cells showed significantly less ability to proliferate against mTg challenge *ex vivo* (Fig. 10*B*). Cells from CD4+CD25+ T cell recipients produced lesser IFN-γ and IL-2, and higher IL-10 and TGF-β1 compared to non-recipient control mice (Fig. 10*C*). Importantly, IL-10 and TGF-β1 responses by T cells from mice that received IL-1β exposed CD4+CD25+ T cells were significantly higher than that produced by cells from mice that received control CD4+CD25+ T cells. These results showed that IL-1β-treated CD4+CD25+ T cells could promote T cell tolerance to self antigen and suppress autoimmune thyroiditis more efficiently than control CD4+CD25+ T cells.

## Discussion

CD8α− DCs from GM-CSF treated mice that produce significantly lower levels of pro-inflammatory cytokines IL-12 and IL-1β compared to the DCs from control mice can induce and/or expand Foxp3+ Tregs and suppress autoimmune diseases [19], [35]. While pro-inflammatory cytokines have been generally associated with the induction of inflammation and autoimmunity, some studies have shown that they may provide positive signals for the induction of Tregs [41], [42]. Several studies have implicated IL-12 and other Th1 cytokines in autoimmune diseases [43], [44], [45], [46]. Recent studies have shown IL-1β as an important mediator of Th17 response in humans and is often associated with autoimmunity [47], [48], [49], [50]. In contrast, IL-1β has also been shown to provide co-stimulation and/or act as a growth factor for Foxp3+ Tregs [42], [51]. Therefore, in the current study we evaluated the effects of IL-12 and IL-1β on Foxp3 expression in T cells that were activated in the presence or absence of CD8α− DCs. Our observations showed that these cytokines have contrasting effects on Foxp3 expression in activated T cells. While the presence of IL-12 in the culture abrogated Foxp3 expression in T cells, IL-1β in the presence of reduced levels of IL-12 promoted Foxp3 expression. These results showed that IL-12 and IL-1β have opposing effects on Tregs. Our results also show that these secreted factors do not significantly modulate DC properties but have a direct effect on T cells.

IL-12 is recognized for its ability to promote Th1 type of pathogenic T cell response in many autoimmune conditions [6], [52] and can overcome immune tolerance by suppressing Foxp3+ Tregs. In a recent study, Brahmachari *et al*. have shown that the p40 subunit of IL-12 can down-regulate Foxp3 expression via the production of nitric oxide [53]. Our observation that IL-12 abrogates Foxp3 expression in T cells during activation further confirms the pro-inflammatory and the potential pathogenic effects of this cytokine in autoimmunity.

IL-1β, on the other hand, has pleiotropic effects and can alter cell signaling, migration and cytokine production, and influence T cell differentiation differently under different conditions [48], [54], [55]. IL-1β has been shown to break peripheral tolerance by facilitating the expansion of effector T cells and is implicated in autoimmune diseases such as rheumatoid arthritis [56]. Lately, IL-1β has been shown to be critical for the generation of IL-17 secreting T helper cells in humans. IL-23 when combined with IL-1β has been shown to induce and maintain pathogenic Th17 cells [47], [50]. Our current finding of a distinct role for IL-1β in promoting Foxp3 expression in T cells is in apparent contradiction to the above findings, but is in agreement with recent observations of Brinster *et al*. who showed that IL-1β expands Foxp3+ Tregs in the presence of anti-CD3 Ab and splenic DCs [51]. In another study, treatment of murine myeloid DCs with rapamycin, an immunosuppressive agent, resulted in the production of IL-1β by phenotypically immature DCs, which were refractive to inflammatory stimuli and thus were unable to activate T cell responses [57]. Moreover, differentiation of DCs from monocytes was impaired when they were pre-treated with low doses of IL-1β, and these DCs were unable to effectively stimulate an immune response [58]. However, our observations failed to show a significant direct modulatory effect of IL-1β on DCs We noted expression of IL-1 receptor transcripts in both DCs and T cells (Figure 1). Recent studies have shown increased expression of IL-1 receptor on Tregs as compared to conventional T cells as well as increased IL-1β signaling in Tregs [2]. Therefore, the higher levels of expression of IL-1 receptors on T cells may be determined by the lineage characteristics of T cells and this may have contributed to the direct effects of IL-1β on T cells noted in this study.

Although nTregs constitutively express Foxp3 during resting stage, expression level of this transcription factor is down-regulated upon *in vitro* activation [59]. This could explain why only less than 50% of CD4+CD25+ T cells expressed Foxp3 after activation in our control cultures. However, our observations indicate that IL-1β, when added to the T cell activation cultures, helps maintain Foxp3 expression in a significantly higher proportion of nTregs upon activation and proliferation (Fig. 4). This suggested that IL-1β may be promoting the expansion of naturally existing CD25+Foxp3+ T cells and/or inducing Foxp3 expression in CD25+Foxp3− effector T cells. The ability of IL-1β to promote higher levels of TGF-β1 production by T cells upon activation suggested that new Foxp3+ T cells could be induced in these cultures. T cells activated in the presence of IL-1β also produced significantly higher levels of IL-2 compared to control T cells. This indicated that these cytokines (i.e. TGF-β1 and IL-2) may have critical roles in inducing and/or maintaining Foxp3 expression in T cells that were activated in the presence of IL-1β.

TGF-β is a pleiotropic cytokine that can facilitate either regulatory or inflammatory responses depending on the levels of other cytokines present in the microenvironment [60]. While TGF-β in combination with IL-2 is responsible for the survival of naïve T cells and helps maintain peripheral tolerance, it is also responsible for the differentiation of pathogenic Th17 cells in association with pro-inflammatory cytokines such as IL-6 [61]. Recent studies have conclusively demonstrated a critical role for IL-2 not only in the homeostasis and functioning of nTregs, but also in the generation of adaptive Tregs [62], [63], [64]. Our observation that neutralization of IL-2 or TGF-β1 can reverse IL-1β-dependent increase in Foxp3+ T cell frequencies in the culture supports the notion that IL-1β promotes TGF-β1 and IL-2 dependent Foxp3 expression in T cells.

The nTregs are not known to produce significant amounts of IL-2 upon activation [65]. Therefore, effector T cells are considered the primary source of IL-2 when total CD4+ T cells are activated using anti-CD3 and anti-CD28 Abs. Importantly, although most nTregs constitutively express high levels of CD25 on the surface, a significant proportion of CD4+CD25+ T cells enriched from normal mice could be Foxp3− activated effector T cells. This would explain why both CD4+CD25+ and CD4+CD25− T populations produced considerable amounts of IL-2 upon activation in our study. Our observation that only CD4+CD25+ T cells, but not CD4+CD25− cells produced TGF-β1 indicated that enriched CD4+CD25+ T cell preparation contains both Tregs and activated effector T cells, and this population, but not nTregs or CD4+CD25− cells, can produce significant amounts both TGF-β1 and IL-2 during activation leading to induction and/or maintenance of Foxp3 expression in them; the exogenous IL-1β enhances this effect.

Studies using purified GFP+Foxp3+ cells from Foxp3-GFP knock-in mice showed little or no proliferation when activated using anti-CD3 and anti-CD28 Abs with or without IL-1β. Although a considerable number of Foxp3+ T cells express TGF-β1 upon activation in the presence of IL-1β, it appears that IL-1β by itself does not promote Foxp3+ T cell proliferation. The inability of Foxp3+ cells to proliferate was most likely due to their failure to produce sufficient amounts of IL-2. In fact earlier studies have demonstrated that Foxp3+ nTregs require high amounts of exogenous IL-2 for their proliferation [66]. In this regard, secretion of much higher amounts of TGF-β1 by CD4+CD25+ T cells, upon activation in the presence of IL-1β, suggested that it likely acted as an autocrine and helped sustain Foxp3 expression, while IL-2 secreted by Foxp3−CD25+ T cells facilitated proliferation of Foxp3+ cells. In fact, a recent study has shown that IL-1β-induced IL-2 production by Foxp3− T cells is essential for IL-1β-mediated expansion of Foxp3 T cells [51].

Very little is known about how IL-1β regulates IL-2 and TGF-β production. While studies have reported a negative correlation between IL-1β and TGF-β [67], [68], [69], Tao Lu *et al*. showed that TGF-β and IL-1β cross activate each other in a dose-dependent manner [70]. Earlier studies have also shown the induction of TGF-β by IL-1β in different human and animal tissues [71], [72], [73]. Moreover, IL-1β has been shown to increase TGF-β1 in articular chondrocytes through the activation of AP-4 and AP-1 binding to the TGF-β1 gene promoter [72]. Therefore, it appears that IL-1β and TGF-β regulate the expression of each other in different immune cells, and that this regulation is dependent upon their absolute and relative concentrations as well as on the presence of other cytokines such as IL-2. Our observation that TGF-β secretion and Foxp3 expression by Tregs is dependent on the concentration of IL-1β as well as presence of IL-2 in the culture supports this notion. While we have no direct evidence on the regulation of IL-2 production by IL-1β, the increased IL-2 production by effector T cells in the presence of IL-1β is likely the result of this cytokine's ability to enhance T cell proliferation upon activation.

Overall, our observations show that IL-1β can 1) enhance the proliferation of effector T cells upon activation, 2) increase the production of IL-2 by these proliferating cells, 3) enhance the activation and proliferation of Foxp3+ cells in the presence of IL-2 from effector T cells, 4) increase the production of TGF-β1 by Foxp3+ cells and 5) promote IL-2 and TGF-β1 dependent Foxp3 expression in both Tregs and effector T cells. The observation that IL-1β-exposed CD4+CD25+ cells can suppress effector T cell response *in vitro* and autoimmune thyroiditis in CBA/j mice suggest that the increase in the frequency of Foxp3+ Tregs in IL-1β-treated cultures may account for the enhanced suppression of autoimmune response when compared to controls. It must be noted that the control Tregs could also suppress autoimmune disease but to a lesser extent.

In conclusion, our study demonstrates a new role for IL-1β in promoting and enhancing Treg function. Considering that this cytokine is also produced during inflammatory responses, it is reasonable to assume that IL-1β has an immunoregulatory role. Based on our observations, we speculate that one of the functions of IL-1β produced during an inflammation may be to promote the suppressor function of Tregs to avoid autoimmune and lymphoproliferative responses.

## Supporting Information

Figure S1 **Purified DCs and T cells, both express IL-R with and without treatment with IL-1β.** Purified CD11c+ DCs and CD4+ T cells were purified from spleens of naïve C57Bl/6 mice using antibodies bound to magnetic beads. To determine IL-1β receptor expression on the surface of T cells and DCs with or without treatment with IL-1β, T cells and DCs were isolated using magnetic beads and cultured in the presence of 5 ng of IL-1β or medium alone for 24 hours. Subsequently, RNA was isolated from these cells and used in a RT-PCR assay to detect cytokine transcripts for IL-R2. Primers to detect HPRT were used as controls. PCR products were collected at different cell cycles and were resolved on a 2% agarose gel. Primer sequences are: HPRT-F-GTTGGATACAGGCCAGACTTTGTTG, HPRT-R-TACTAGGCAGATGGCCAGGACTA; IL-1R2F-TGCAAAGTGTTTCTGGGAAC, IL-1R2R-ATATTGCCCCCACAACCAAG.
(TIF)
