# Supplementary figures and images for: IL-1β Promotes TGF-β1 and IL-2 Dependent Foxp3 Expression in Regulatory T Cells
Source: PLoS One. 2011 Jul 11;6(7):e21949. doi: 10.1371/journal.pone.0021949 (PMC3136935; doi:10.1371/journal.pone.0021949)

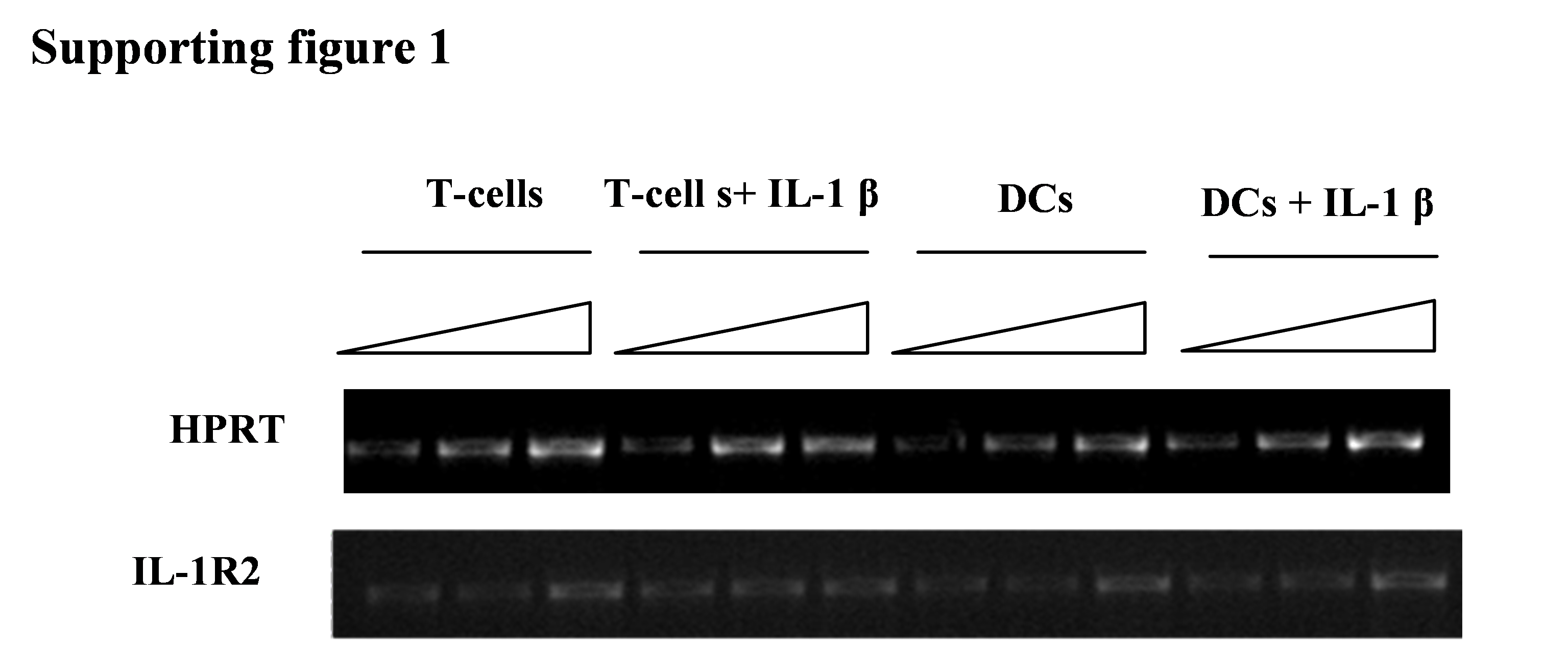

Supplement: Figure S1 — Purified DCs and T cells, both express IL-R with and without treatment with IL-1β. Purified CD11c+ DCs and CD4+ T cells were purified from spleens of naïve C57Bl/6 mice using antibodies bound to magnetic beads. To determine IL-1β receptor expression on the surface of T cells and DCs with or without treatment with IL-1β, T cells and DCs were isolated using magnetic beads and cultured in the presence of 5 ng of IL-1β or medium alone for 24 hours. Subsequently, RNA was isolated from these cells and used in a RT-PCR assay to detect cytokine transcripts for IL-R2. Primers to detect HPRT were used as controls. PCR products were collected at different cell cycles and were resolved on a 2% agarose gel. Primer sequences are: HPRT-F-GTTGGATACAGGCCAGACTTTGTTG, HPRT-R-TACTAGGCAGATGGCCAGGACTA; IL-1R2F-TGCAAAGTGTTTCTGGGAAC, IL-1R2R-ATATTGCCCCCACAACCAAG. (TIF) [file pone.0021949.s001.tif]
